# Polymorphisms of the *UCP2* Gene Are Associated with Glomerular Filtration Rate in Type 2 Diabetic Patients and with Decreased *UCP2* Gene Expression in Human Kidney

**DOI:** 10.1371/journal.pone.0132938

**Published:** 2015-07-28

**Authors:** Bianca Marmontel de Souza, Marcus Michels, Denise Alves Sortica, Ana Paula Bouças, Jakeline Rheinheimer, Marjoriê Piuco Buffon, Andrea Carla Bauer, Luís Henrique Canani, Daisy Crispim

**Affiliations:** 1 Endocrine Division, Hospital de Clínicas de Porto Alegre, Porto Alegre, Rio Grande do Sul, Brazil; 2 Postgraduate Program in Medical Sciences: Endocrinology, Universidade Federal do Rio Grande do Sul, Porto Alegre, Rio Grande do Sul, Brazil; Democritus University of Thrace, GREECE

## Abstract

**Introduction:**

Uncoupling protein 2 (UCP2) reduces production of reactive oxygen species (ROS) by mitochondria. ROS overproduction is one of the major contributors to the pathogenesis of chronic diabetic complications, such as diabetic kidney disease (DKD). Thus, deleterious polymorphisms in the *UCP2* gene are candidate risk factors for DKD. In this study, we investigated whether *UCP2* -866G/A, Ala55Val and Ins/Del polymorphisms were associated with DKD in patients with type 2 diabetes mellitus (T2DM), and whether they had an effect on *UCP2* gene expression in human kidney tissue biopsies.

**Materials and Methods:**

In a case-control study, frequencies of the *UCP2* -866G/A, Ala55Val and Ins/Del polymorphisms as well as frequencies of the haplotypes constituted by them were analyzed in 287 T2DM patients with DKD and 281 T2DM patients without this complication. In a cross-sectional study, *UCP2* gene expression was evaluated in 42 kidney biopsy samples stratified according to the presence of the *UCP2* mutated -866A/55Val/Ins haplotype.

**Results:**

In the T2DM group, multivariate logistic regression analysis showed that the -866A/55Val/Ins haplotype was an independent risk factor for DKD (OR = 2.136, 95% CI 1.036–4.404), although neither genotype nor allele frequencies of the individual polymorphisms differed between case and control groups. Interestingly, T2DM patients carrying the mutated haplotype showed decreased estimated glomerular filtration rate (eGFR) when compared to subjects with the reference haplotype (adjusted P= 0.035). In kidney biopsy samples, *UCP2* expression was significantly decreased in *UCP2* mutated haplotype carriers when compared to kidneys from patients with the reference haplotype (0.32 ± 1.20 vs. 1.85 ± 1.16 n fold change; adjusted P< 0.000001).

**Discussion:**

Data reported here suggest that the *UCP2* -866A/55Val/Ins haplotype is associated with an increased risk for DKD and with a lower eGFR in T2DM patients. Furthermore, this mutated haplotype was associated with decreased *UCP2* gene expression in human kidneys.

## Introduction

Diabetic kidney disease (DKD), also known as diabetic nephropathy, is a major chronic complication of diabetes mellitus (DM) and the leading cause of end-stage renal disease (ESRD) that requires dialysis treatment or kidney transplantation [1,2]. This complication affects approximately 40% of type 2 DM (T2DM) patients, and is an important cause of morbidity and mortality among these subjects [2,3,4]. Usually, DKD is a progressive disorder characterized by pathophysiological alterations resulting from the diabetic state, which begin with glomerular hyperfiltration and renal hypertrophy, and might progress to proteinuria and a gradual decrease in glomerular filtration rate (GFR) [1,4].

Although hyperglycemia, arterial hypertension and dyslipidemia are known risk factors for DKD, a subset of subjects with poorly controlled DM does not develop this complication, indicating that genetic factors might have a key role in its pathogenesis [5]. In fact, several studies have shown that genetic susceptibility contributes to the development of DKD in both type 1 and type 2 DM [6,7,8,9]. Therefore, great efforts have been made to identify genetic variants associated with DKD; however, results are still inconclusive with different variants associated with small effects in different populations [1].

It is well known that hyperglycemia causes an important increase in the production of reactive oxygen species (ROS) by mitochondria [6,10]. In this context, Du *et al*. [11] proposed a unifying hypothesis linking important pathways involved in the pathogenesis of DKD. Accordingly to this hypothesis, hyperglycemia-induced mitochondrial superoxide overproduction results in an increased activation of protein kinase C isoforms, increased formation of advanced glycation end-products (AGE), acceleration of glucose flux through the aldose-reductase pathway, and an increased glucose flux into the hexosamine pathway. These alterations stimulate growth factors that result in extracellular matrix accumulation, leading to DKD.

Uncoupling protein 2 (UCP2) belongs to an anion-carrier protein family located in the mitochondrial inner membrane [12,13], and it is expressed in many tissues, including white adipose tissue, pancreatic islets, retinal cells and kidneys [14,15,16,17]. UCP2 mildly uncouples substrate oxidation from ATP synthesis, thereby dissipating the membrane potential energy and, consequently, decreasing ATP production by mitochondrial respiratory chain [15,18]. The uncoupling thus leads to tissue-specific functions such as regulation of free fatty acid metabolism, inhibition of insulin secretion from pancreatic beta-cells and, importantly, decreasing ROS formation by mitochondria [12,17]. Thus, polymorphisms in the *UCP2* gene might be involved in the development of DKD or other diabetic chronic complications.

The *UCP2* gene covers an 8,174 kb region on chromosome 11q13, and it has eight exons and seven introns (http://genome.ucsc.edu). The transcriptional gene unit comprises two untranslated exons followed by six exons that encode the 309 amino acids of the UCP2 protein [19]. So far, most identified polymorphisms in the *UCP2* gene have shown low frequencies and have therefore not been so intensively studied. However, there are 3 common *UCP2* polymorphisms, which are well studied: the -866G/A polymorphism (rs659366) in the promoter region, the Ala55Val polymorphism (rs660339) in exon 4, and the 45 bp insertion/deletion (Ins/Del) polymorphism in the 3’ untranslated region (3’UTR) [20,21].

Taking into consideration the role of UCP2 in the protection against oxidative stress, our group previously investigated whether the *UCP2* -866G/A, Ala55Val and Ins/Del polymorphisms, also described in association with T2DM, could be also associated with diabetic retinopathy (DR) [22]. Our data showed that the -866A/55Val/Ins haplotype was associated with increased risk for proliferative DR in both type 1 and type 2 DM patients. More recently, we evaluated if the -866A/55Val/Ins haplotype was associated with changes in *UCP2* gene expression in retina from cadaveric cornea donors. Interestingly, carriers of the mutated haplotype showed a lower *UCP2* gene expression in retina than homozygous for the reference haplotype (-886G/55Ala/Del) [23].

Therefore, in this study, we investigated whether the *UCP2* -866G/A, Ala55Val and Ins/Del polymorphisms were associated with DKD in T2DM patients, and whether they had an effect on *UCP2* gene expression in human kidney tissue biopsies.

## Materials and Methods

### T2DM patients and phenotype measurements

A total of 568 unrelated T2DM patients were enrolled in the study. The sample population comprised 287 T2DM patients with DKD (cases) and 281 T2DM patients without this complication and with known DM duration of at least 10 years (controls). T2DM patients were participating in a multicenter study that recruited patients in Southern Brazil from 2002 to 2013. That project was designed to study genetic risk factors associated with T2DM and its chronic complications, such as DKD and DR. It initially had four participating centers located in teaching hospitals in the Brazilian State of Rio Grande do Sul, specifically Grupo Hospitalar Conceição, Hospital São Vicente de Paula, Hospital Universitário de Rio Grande, and Hospital de Clínicas de Porto Alegre. A detailed description of the study can be found elsewhere [24]. T2DM was defined as a diagnosis of DM after the age of 35 years, with no insulin therapy during the first year after diagnosis and no previous episodes of ketoacidosis [25]. The ethnic group was defined based on self-classification, and the ethnic proportion between case and controls was as follows: 21.9% of black patients in the case group and 21.3% of black patients in the control group (P = 0.860).

A standard questionnaire was used to collect information about age, age at T2DM diagnosis, and drug treatment. All T2DM patients underwent physical and laboratory evaluations, as previously described [22,26]. Briefly, they were weighed bare feet, wearing light outdoor clothes and their height was measured. Body mass index (BMI) was calculated as weight (kg)/height (meters)^2^. Office blood pressure (BP) was measured in sitting position, on the left arm, after a 5-min rest by a trained nurse, with a mercury sphygmomanometer. The mean of two measurements taken 1 min apart was used to calculate systolic and diastolic BP. Arterial hypertension (AH) was defined as BP levels ≥140/90 mmHg at the initial visit and at two follow-up visits within 1 month of the initial visit, or if the presence of AH was previously registered on medical records.

The diagnosis of DKD was primarily based on the albumin excretion rate (AER) in at least two out of three consecutive 24-h timed urine samples in a 6-month period. Patients were classified as having normal to mildly increased AER (AER < 30 mg/24h, **control group**), moderately increased AER (AER 30–299 mg/24h) or severely increased AER (AER ≥ 300 mg/24h) [27]. Therefore, the **case group** with DKD was constituted by patients having moderately to severely increased AER. Patients with other causes of albuminuria or renal diseases other than DKD were excluded. Moreover, independently of AER, patients were also evaluated regarding their estimated GFR (eGFR) [27]. Estimated GFR was calculated using the Modification of Diet in Renal Disease (MDRD) equation (http://nephron.org/mdrd_gfr_si), which takes into account the following parameters: age, gender, ethnicity and creatinine value. According to the MDRD equation, eGFR values above 60 ml/min/1.73m^2^ should be interpreted as “above 60 ml/min/1.73m^2^”, not an exact number. An experienced ophthalmologist assessed all patients for DR using fundoscopy through dilated pupils [28].

Serum and plasma samples were taken after 12 hours of fasting for laboratory analyses. Glucose levels were determined using the glucose oxidase method. HbA1c measurements were performed by different methods and the results were traceable to the Diabetes Control and Complications Trial (DCCT) method by off-line calibration or through conversion formulae [29]. Creatinine was measured by the Jaffé reaction; total plasma cholesterol, HDL cholesterol and triglycerides by enzymatic methods, and albuminuria by immunoturbidimetry (Sera-Pak immuno microalbuminuria, Bayer, Tarrytown, NY, USA; mean intra and interassay coefficients of variance of 4.5% and 11% respectively) [30]. Patients interrupted the use of angiotensin-converting enzyme inhibitors or angiotensin receptor antagonists for at least one week before having their albuminuria measured.

The study protocol was approved by Ethic Committee in Research from Hospital de Clínicas de Porto Alegre and all patients gave their informed consent in writing. Clinical investigation has been conducted according to the principles expressed in the Declaration of Helsinki.

### Kidney samples and phenotype measurements

To investigate *UCP2* gene expression in the presence of different *UCP2* haplotypes, kidney biopsies were obtained from 118 patients who underwent therapeutic nephrectomy suggested by an urologist in Hospital de Clínicas de Porto Alegre. A standardized form was used to collect information from medical records about age, sex, presence of AH and DM, smoking habits, and occurrence of other diseases. Peripheral blood samples were collected from each subject for DNA extraction and genotyping of the *UCP2* polymorphisms of interest. Following genotyping, subjects were divided into groups according to the presence of the *UCP2* mutated haplotype (-866A/55Val/Ins).

Most of subjects had their kidney removed due to malignant disease. After nephrectomy, an excised normal kidney biopsy was snap-frozen in liquid nitrogen and stored at -80°C until *UCP2* mRNA expression analysis. Only kidney samples containing normal tissue without visible tumors at optical microscopy were eligible for inclusion in the study. The study protocol was approved by Ethic Committee in Research from Hospital de Clínicas de Porto Alegre and all patients gave their informed consent in writing. Clinical investigation has been conducted according to the principles expressed in the Declaration of Helsinki.

### Genotyping

DNA was extracted from peripheral blood leucocytes by a standardized salting-out procedure. The *UCP2* -866G/A polymorphism (rs659366) was determined by digesting polymerase chain reaction (PCR) products with the restriction enzyme *MluI* (Invitrogen Life Technologies, Inc., CA, USA), as previously described [23]. Digestion fragments were resolved on 2% agarose gels containing GelRed Nucleic Acid Gel Stain (Biotium, Inc., Hayward, CA) and visualized under ultraviolet illumination. A sample of DNA (whose genotype was identified by sequencing) was used as a positive control to evaluate the completeness of PCR product digestion. Evaluation of the *UCP2* 45 bp Ins/Del polymorphism was done by PCR, as previously described [23]. Briefly, primers amplified products of 457 bp (insertion allele) or 412 bp (deletion allele), which were resolved on 2.5% agarose gels stained with GelRed Nucleic Acid Gel Stain (Biotium, Inc.) and visualized under ultraviolet light. Genotypes of the -866G/A and Ins/Del polymorphisms were recorded using the ImageMaster System VDS (GE HealthCare, London, UK).

Genotyping of the *UCP2* Ala55Val (C/T) polymorphism (rs660339) was determined using primers and probes contained in the Human Custom TaqMan Genotyping Assay 40x (Life Technologies, Foster City, CA, USA). Primer and probe sequences can be found elsewhere [23].

### RNA isolation

Kidney tissue biopsies (250 mg) were homogenized in phenol-guanidine isothiocyanate (Invitrogen—Life Technologies). RNA was extracted with chloroform and precipitated with isopropanol by centrifugation (12,000 x g) at 4°C. RNA pellet was washed twice with 75% ethanol and ressuspended in 10–50 μL of diethylpyrocarbonate treated water.

Concentration and quality of total RNA samples were assessed using a NANODROP 2000 spectrophotometer (Thermo Scientific Inc., DE, USA). Only RNA samples which achieved adequate purity ratios (A260/A280 = 1.9–2.1) were used for subsequent analyses [31]. In addition, RNA integrity and purity were also checked on agarose gel containing GelRed Nucleic Acid Gel Stain (Biotium, Inc.). The mean RNA concentration (± SD) isolated was 16.8 ± 31.4 μg / 250 mg kidney tissue biopsy.

### Quantification of *UCP2* gene expression by Real-Time qPCR

Real-time reverse transcription-PCR was performed in two separate reactions: first, RNA was reverse transcribed into cDNA, then cDNA was amplified by quantitative real-time PCR (RT-qPCR). Reverse transcription of 2.5 μg of RNA into cDNA was carried out using the SuperScript VILO Master Mix for RT-PCR (Invitrogen—Life Technologies), following the manufacturer’s protocol for the random primer method.

RT-qPCR experiments were performed in a 7500 Fast Real-Time PCR System Thermal Cycler (Life Technologies). Experiments were performed by monitoring in real-time the increase in fluorescence of the SYBER Green dye [32]. Primers for *UCP2* and *GAPDH* genes were designed using published human gene sequences and the Primer Express 3.0 Software (Life Technologies), and they were projected to target two consecutive exons of a gene in order to prevent the amplification of any contaminating genomic DNA. Primer sequences were as follows: *UCP2* F 5’-TTGGGTTCAAGGCCACAGAT-3’, *UCP2* R 5’-CCAGCCCCAAGAAACTTCAC-3’, *GAPDH* F 5’-ACCCACTCCTCCACCTTTG-3’, and *GAPDH* R 5’-CTCTTGTGCTCTTGCTGGG-3’.

PCR reactions were performed using 10 μL of 2x Fast SYBER Green Master Mix (Life Technologies), 1 μL (1 ng/μL) of forward and reverse primers for *UCP2* or *GAPDH* and 1 μL of cDNA template (1.25 μg/μL), in a total volume of 20 μL and following PCR conditions described elsewhere [23].

Quantification of *UCP2* mRNA was performed by relative quantification using the comparative ΔΔCq method [31,33], and expressed relative to the reference gene (*GAPDH*). Validation assays were done by amplification of the target (*UCP2*) and reference (*GAPDH*) genes, separately, using serial dilutions of a pool of six kidney cDNA samples. As a requirement of this method, both target and reference genes exhibited equal amplification efficiencies (*E* = 95–105%; curve slope: *UCP2* = 3.409, *GAPDH* = 3.444) in all experiments. The ΔΔCq method calculates changes in gene expression as relative fold differences (n-fold changes) between an experimental and an external calibrator sample [31,33].

Among the initial 118 kidney biopsy samples genotyped for the three *UCP2* polymorphisms, only 42 samples were selected for *UCP2* gene expression analysis: 15 carrying the reference haplotype (-866G/55Ala/Del) in homozygosis, 15 heterozygous, and 12 carrying the mutated haplotype (-866A/55Val/Ins) in homozygosis. These numbers were calculated in order to be sufficient to detect a 0.5 n fold difference between groups (beta = 80%, α = 0.05).

### Statistical analyses

Allele frequencies were determined by gene counting and deviations from the Hardy-Weinberg equilibrium (HWE) were verified using the χ^2^ test. Allele and genotype frequencies were compared between groups using the χ^2^ test. The haplotypes constructed from the combination of the three *UCP2* polymorphisms and their frequencies were inferred using the PHASE 2.1 program, which implements a Bayesian statistical method [34].

Clinical and laboratory characteristics and *UCP2* mRNA concentrations were compared between groups by using unpaired Student’s t-test, one-way ANOVA or χ^2^ test, as appropriate. Variables with normal distribution are presented as mean ± SD or percentage. Variables with a skewed distribution were logarithmically transformed before analyses and are presented as median (minimum–maximum values) or mean (95% CI).

The magnitude of the association of different *UCP2* polymorphisms or haplotypes with DKD was estimated using odds ratio (OR) tests with 95% CI. Multivariate logistic regression analyses were performed to assess the independent association of individual *UCP2* polymorphisms or haplotypes with DKD, as well as to control for possible confounding factors whenever a statistically significant association was found in univariate analyses. DM duration was not included as an independent variable in these analyses because the control group (without DKD) was selected based on this feature. Multiple linear regression analysis was performed with eGFR (logarithmic) as a dependent variable and age, gender, AH, T2DM duration, and the presence of the mutated *UCP2* haplotype as independent variables. Moreover, linear regression analysis was performed with *UCP2* gene expression (logarithmic) as dependent variable and age, gender, diagnosis of DM and presence of the *UCP2* mutated haplotype as independent variables. Pearson’s correlation test was used to assess correlations between different quantitative variables. A *P* value of <0.05 was considered statistically significant. These statistical analyses were done with SPSS version 18.0 (SPSS, Chicago, IL, USA).

## Results

### Study of the association between *UCP2* polymorphisms and DKD

As expected, T2DM patients with DKD differed significantly from control patients for gender, T2DM duration, HDL cholesterol, triglycerides and creatinine levels, and occurrence of DR (**Table 1**). Frequencies of the *UCP2* -866G/A, Ala55Val and Ins/Del genotypes did not differ between white and black T2DM patients (all P values > 0.300). Neither genotype nor allele frequencies of the -866G/A, Ala55Val and Ins/Del polymorphisms differed statistically between cases with DKD and controls without this complication (**Table 2**), and all genotypes were in agreement with those predicted by the HWE in all groups (P > 0.05). Frequencies of mutated allele carriers (dominant model) were also similar between groups, and the adjustment for covariables did not change these results (**Table 2**). It is worth mentioning that these polymorphisms remained not associated with DKD when taking into account recessive or additive inheritance models (data not shown). Moreover, frequencies of the analyzed *UCP2* polymorphisms were not significantly different between patients with moderately or severely increased AER (P ≥ 0.20, data not shown).

**Table 1:** Clinical and laboratory characteristics of T2DM patients broken down by the presence of DKD.

	Control group (n = 281)	Case group (n = 287)	P* value
Age (years)	61.4 ± 9.6	60.2 ± 10.2	0.281
Gender (% males)	34.2	56.4	< 0.000001
Ethnicity (% black)	21.9	21.3	0.860
T2DM duration (years)	16.5 ± 6.5	14.7 ± 9.1	0.006
BMI (kg/m^2^)	28.4 ± 4.7	29.0 ± 5.1	0.153
HbA1c (%)	6.99 ± 1.98	6.88 ± 2.12	0.537
Systolic BP (mmHg)	142.5 ± 22.3	144.8 ± 22.6	0.226
Diastolic BP (mmHg)	85.8 ± 13.1	86.3 ± 13.4	0.669
Total cholesterol (mg/dL)	208.9 ± 45.0	213.8 ± 49.1	0.275
HDL cholesterol (mg/dL)	46.1 ± 12.1	42.9 ± 12.6	0.002
Triglycerides (mg/dL)	145 (35–892)	164 (44–1470)	0.005
Diabetic retinopathy (%)	42.8	66.2	< 0.000001
Creatinine (μg/dL)	0.9 (0.5–2.99)	1.1 (0.4–13.6)	< 0.000001

Data are mean ± SD, median (minimum-maximum values) or %. BMI, body mass index; BP, blood pressure; HbA1c, glycated hemoglobin; T2DM, type 2 diabetes mellitus.

*P values are according to χ^2^ test or t-test as appropriate.

**Table 2:** Genotype and allele distributions of *UCP2* polymorphisms in T2DM patients with and without DKD.

*UCP2* polymorphisms	Control group	Case group	Unadjusted P value*	Adjusted OR (95% CI) / P value§
**-866 (G/A)**	n = 278	n = 287		
G/G	99 (35.6)	101 (35.2)	0.965	1
G/A	131 (47.1)	134 (46.7)		0.763 (0.446–1.304) / 0.322
A/A	48 (17.3)	52 (18.1)		0.825 (0.424–1.607) / 0.572
G	0.592	0.585	0.875	-
A	0.408	0.415		
*Dominant Model*				
G/G	99 (35.6)	101 (35.2)	0.987	1
G/A + A/A	179 (64.4)	186 (64.8)		0.780 (0.471–1.293) / 0.336
**Ala55Val (C/T)**	n = 281	n = 287		
Ala/Ala	93 (33.1)	102 (35.5)	0.828	1
Ala/Val	135 (48.0)	133 (46.3)		0.700 (0.407–1.202) / 0.196
Val/Val	53 (18.9)	52 (18.2)		0.793 (0.408–1.542) / 0.494
Ala	0.571	0.587	0.629	-
Val	0.429	0.413		
*Dominant Model*				
Ala/Ala	93 (33.1)	102 (35.5)	0.600	1
Ala/Val + Val/Val	188 (66.9)	185 (64.5)		0.726 (0.436–1.209) / 0.219
**45 bp Ins/Del**	n = 278	n = 287		
Del/Del	132 (47.5)	144 (50.5)	0.181	1
Ins/Del	124 (44.6)	110 (38.3)		0.753 (0.459–1.235) / 0.261
Ins/Ins	22 (7.9)	33 (11.5)		1.218 (0.539–2.752) / 0.636
Del	0.698	0.700	0.978	-
Ins	0.302	0.300		
*Dominant model*				
Del/Del	132 (47.5)	144 (50.2)	0.578	1
Ins/Del + Ins/Ins	146 (52.5)	143 (49.8)		0.822 (0.513–1.317) / 0.415
**Presence of *UCP2* mutated haplotype**	n = 278	n = 287		
*Dominant model* ^a^				
Other haplotypes	141 (50.7)	145 (50.5)	0.963	1
-866A/55Val/Ins	137 (49.3)	142 (49.5)		0.816 (0.498–1.336) / 0.418
*Recessive model* ^b^				
Other haplotypes	260 (93.5)	255 (88.9)	0.071	1
-866A/55Val/Ins -866A/55Val/Ins	18 (6.5)	32 (11.1)		2.136 (1.036–4.404) / 0.040

Data are presented as number of carriers (%) or proportion.

*P values were computed using χ² tests to compare control (T2DM patients without DKD and with more than 10 years of DM duration) and case (T2DM patients with DKD) groups.

§ Adjusted OR (95% CI) / P values adjusted for age, gender, treatment with ACE-inhibitors, triglycerides levels, and eGFR (logarithmic scale) in logistic regression analyses.

^a^ Presence of the mutated -866A/55Val/Ins haplotype (homozygosis + heterozygosis)

^b^ Mutated haplotype in homozygosis *vs*. other haplotypes.

As already described, the -866G/A polymorphism is in almost complete linkage disequilibrium (LD) with the Ala55Val polymorphism (|D’| = 0.991, *r* ^2^ = 0.905), but only in moderate LD with the Ins/Del polymorphism (|D’| = 0.855, *r* ^2^ = 0.485) in our population [22]. The Ala55Val polymorphism is also in partial LD with the Ins/Del polymorphism (|D’| = 0.878, *r* ^2^ = 0.471). Seven haplotypes produced by the combination of the -866G/A, Ala55Val and Ins/Del polymorphisms were inferred in the total sample of T2DM patients. Haplotypes -866G/55Ala/Del (reference; 52.5%), -866A/55Val/Del (13.0%) and -866A/55Val/Ins (mutated; 25.7%) were inferred in frequencies higher than 5% and altogether accounted for 91.2% of the observed haplotypes, with the remaining 8.8% being shared among haplotypes -866G/55Ala/Ins, -866G/55Val/Del, -866G/55Val/Ins, and -866A/55Ala/Del. Taking into consideration the results of our previous study showing that the mutated -866A/55Val/Ins haplotype was associated with increased risk for proliferative DR [22], only subjects carrying the mutated -866A/55Val/Ins haplotype (homozygosis/heterozygosis) or the reference -866G/55Ala/Del haplotype were selected for subsequent analyses. Of note, frequencies of the mutated haplotype were similar between white and black T2DM patients: frequencies of the mutated haplotype in a recessive model = 9.6% in white patients *vs*. 8.4 in black patients (P = 0.702); frequencies in a dominant model = 50.3% in white vs. 48.9% in black patients (P = 0.762).

The frequency of the mutated haplotype (recessive model) was higher in patients with DKD (11.5%) as compared to control patients (6.5%); however, this difference did not reach formal statistical significance (P = 0.071). Interestingly, after adjusting for age, gender, treatment with ACE-inhibitors, triglycerides levels and eGFR, homozygosis for the mutated haplotype was statistically associated with risk for DKD (OR = 2.136, 95% CI 1.036–4.404; **Table 2**).

**Fig 1:**
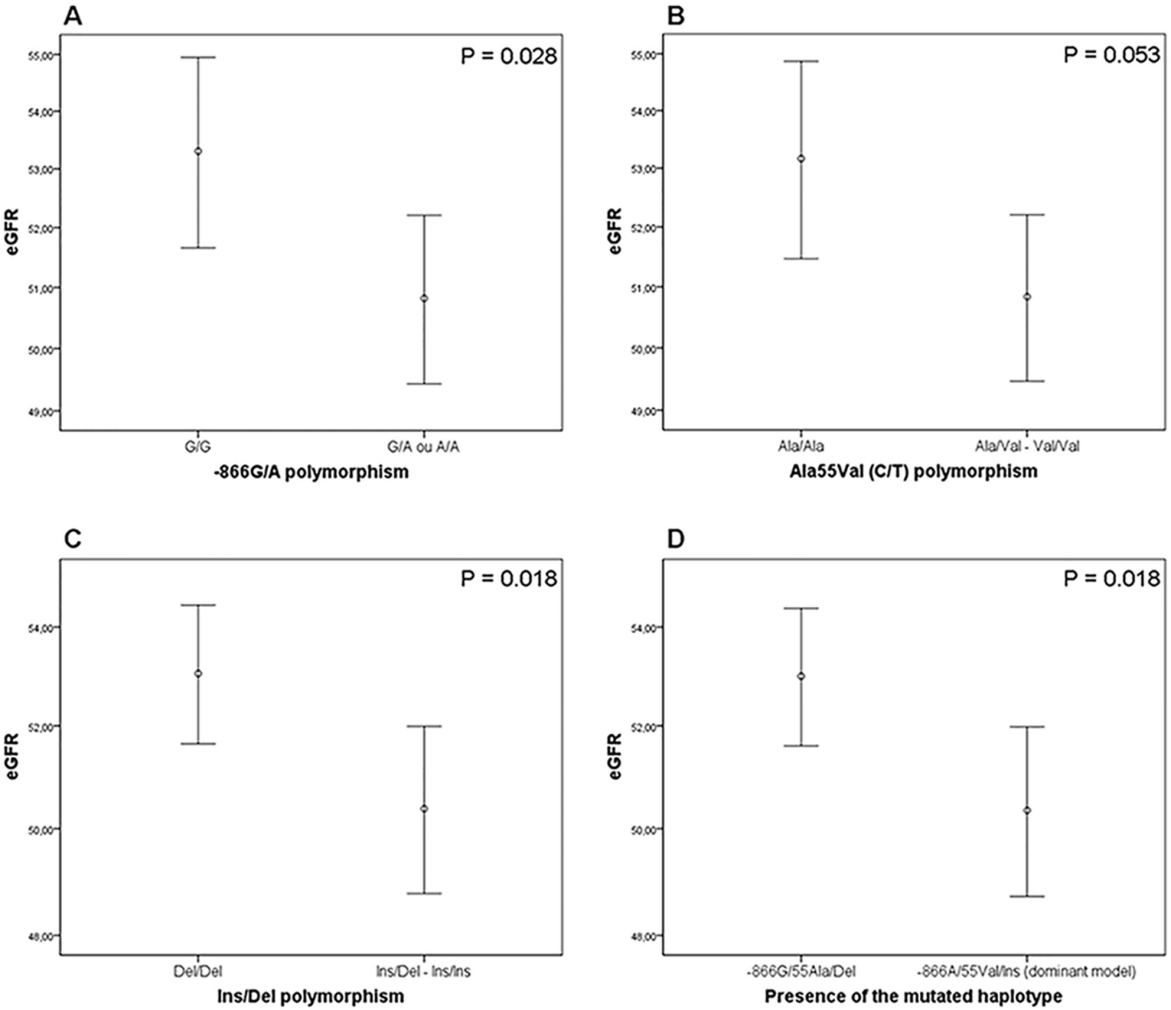
Estimated glomerular filtration rate (eGFR) in T2DM patients according *UCP2* polymorphisms and mutated *UCP2* haplotype. **A)** eGFR in patients stratified according to the presence of the A allele of the -866G/A polymorphism (dominant model). **B)** eGFR in patients stratified according to the presence of the Val allele of the Ala55Val polymorphism (dominant model). **C)** eGFR in patients according to the presence of the Ins allele of the Ins/Del polymorphism (dominant model). **D)** eGFR in patients according to the presence of the *UCP2* mutated haplotype (-866A/55Val/Ins; dominant model). P values were obtained using Student’s t-tests. Data are presented as mean (95% CI).

Interestingly, T2DM patients carrying the minor alleles of the analyzed *UCP2* polymorphisms showed decreased eGFR when compared to subjects homozygous for the reference genotypes (**Fig 1**). Accordingly, patients carrying the mutated haplotype (dominant model) showed lower eGFR compared to subjects with the reference haplotype (P = 0.018; **Fig 1**), and this difference remained statistically significant after adjusting for age, gender, AH and T2DM duration (β = -2.231, P = 0.035).

### *UCP2* gene expression in human kidney biopsies according to the presence of the -866A/55Val/Ins haplotype

*UCP2* gene expression was analyzed in 42 human kidney biopsies collected from 15 patients homozygous for the *UCP2* reference haplotype (-866G/55Ala/Del), 15 heterozygous, and 12 homozygous for the *UCP2* mutated haplotype (-866A/55Val/Ins). The main clinical characteristics of this group were as follows: mean age was 58.3 ± 1.2 years, men comprised 45.2% (n = 19) of the sample, 57.1% (n = 24) of all patients had AH, and 19.0% (n = 8) had DM.

The mean ± SD *UCP2* mRNA concentration in the whole kidney tissue group was 0.88 ± 1.39 n fold change (logarithmic scale). No significant difference was observed when *UCP2* gene expression was analyzed by gender (men: 0.84 ± 1.59 *vs*. women: 0.91 ± 1.21 n fold change; P = 0.879), AH status (normotensive: 0.59 ± 1.57 *vs*. hypertensive: 0.97 ± 1.31 n fold change; P = 0.442), or presence of DM (non-diabetic patients: 0.76 ± 1.44 *vs*. DM patients: 0.93 ± 1.30; P = 0.762 n fold change). *UCP2* gene expression did not correlated with age (r^2^ = -0.054, P = 0.739).

**Fig 2:**
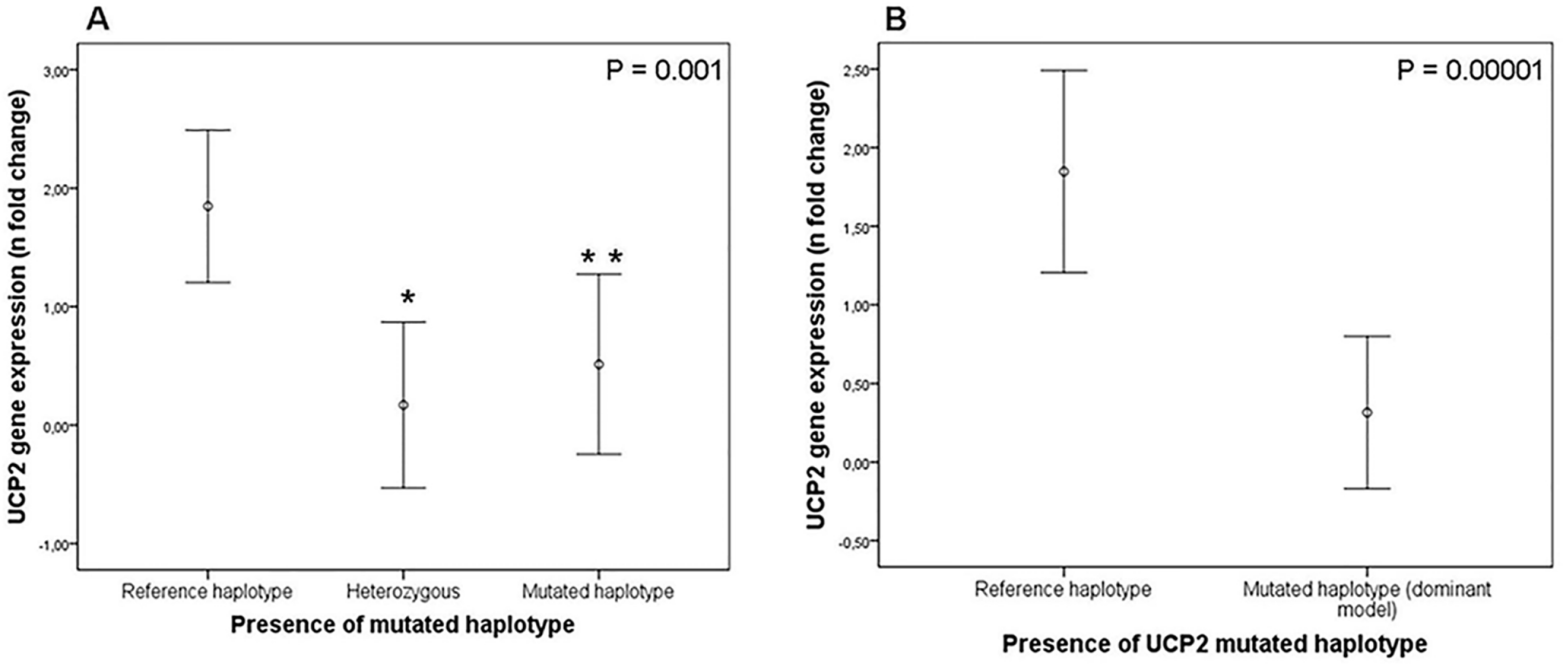
*UCP2* gene expression in human kidney biopsies according the presence of the *UCP2* mutated haplotype. **A)** *UCP2* gene expression in subjects homozygous for the reference haplotype (-866G/55Ala/Del), heterozygous (reference/mutated haplotypes) or homozygous for the mutated haplotype (-866A/55Val/Ins). P value was obtained using One-Way ANOVA test. * P = 0.001 (post-hoc Tukey’s test). ** P = 0.02 (post-hoc Tukey’s test). **B)** *UCP2* gene expression in patients stratified according to the presence of the *UCP2* mutated haplotype (dominant model). P value was obtained using Student’s t-tests. Data are presented as mean (95% CI) of *UCP2* gene expression in logarithmic scale.

*UCP2* gene expression in kidney samples stratified by the presence of the selected *UCP2* haplotypes is depicted in **Fig 2**. *UCP*2 gene expression was decreased in kidney tissue from carriers of the mutated *UCP2* haplotype when compared to kidneys from patients with the reference haplotype (0.32 ± 1.20 *vs*. 1.85 ± 1.16 n fold change, respectively; P < 0.0000001). *UCP2* gene expression was similar between patients heterozygous or homozygous for the mutated haplotype (P = 0.750 from Tukey’s post hoc test; **Fig 2**). After linear regression analysis, the presence of the mutated haplotype remained significantly associated with decreased *UCP2* gene expression after controlling for age, gender and presence of DM (β = -1.913, P < 0.00001).

## Discussion

In the present study, we investigated the frequencies of the *UCP2* -866G/A, Ala55Val and Ins/Del polymorphisms in a sample of T2DM patients according to presence/absence of DKD. Homozygosis for the -866A/55Val/Ins haplotype was associated with risk for DKD after adjustment for covariables. Furthermore, the minor alleles of the analyzed *UCP2* polymorphisms as well as presence of the mutated haplotype were associated with lower eGFR when compared to subjects homozygous for reference genotypes or -866G/55Ala/Del haplotype. It is worth mentioning that the frequencies of the three *UCP2* polymorphisms in our T2DM sample were similar to frequencies observed in other populations [reviewed in [35]].

Although UCP2 plays an acknowledged role in protection against oxidative stress [36], and although oxidative stress is one of the major contributors to the pathogenesis of chronic diabetic complications [10], only a few studies have evaluated the association between *UCP2* polymorphisms and DKD or related phenotypes. Rudofsky *et al*. [37] reported that German T1DM patients carrying the -866A allele had reduced prevalence of diabetic peripheral neuropathy (DPN) when compared with patients with the G/G genotype; however, they did not find any association between the -866G/A polymorphism and DKD or DR, which could be explained by the small sample number analyzed (n = 227). Rudofsky *et al*. [38] studying T2DM patients from Germany also did not observe any association between -866G/A polymorphism and DKD, DR or DPN. In addition, Lindholm *et al*. [39] reported that Ins/Del polymorphism was not associated with DKD in 434 T2DM patients from Scandinavia. Tripathi *et al*. [40] reported a significant association between the Ins/Del polymorphism and risk for ESRD (OR = 8.856; 95% CI 3.458–22.667) in subjects from North India; nevertheless, this result should be interpreted with caution since genotype distributions of this polymorphism were not in HWE in the control group. None of these studies evaluated the association between *UCP2* polymorphisms and eGFR. Further studies are urgently needed to evaluate the association between *UCP2* polymorphisms and DKD and related features in other populations.

Functional polymorphisms can influence gene expression and regulate the final quantity of protein in a given tissue. Therefore, in this study, we also demonstrated that human kidney biopsy samples from patients carrying the mutated *UCP2* -866A/55Val/Ins haplotype, in heterozygosis or homozygosis, showed a 5-fold decrease in *UCP2* gene expression when compared to kidneys from patients with the reference haplotype. This finding is biologically plausible since both -866G/A and Ins/Del polymorphisms have been reported as functional polymorphisms [23,41,42,43,44,45].

In humans, the *UCP2* -866A allele has been reported as being associated with either increased [41,42] or decreased [23,44,45] *UCP2* mRNA levels. A possible explanation for these conflicting results is that this polymorphism seems to be involved in putative binding sites for specific transcription factors [42]. Thus, preferential binding of some transcriptional factor to the G or A allele in the *UCP2* promoter could confer tissue-specific advantages to either allele [42]. As already mentioned, the Ins/Del polymorphism is located in the 3´UTR of the *UCP2* gene. It is well known that 3’UTR is the main site for ligation of microRNAs (miRNAs), which are recognized as major regulators of gene expression. Therefore, it is reasonable to speculate that the Ins/Del polymorphism could be involved in a ligation site for a given miRNA, altering *UCP2* gene expression. Hence, we used the TargetScan software (http://www.targetscan.org; release 6.2) to search for predict miRNAs targeting the human *UCP2* mRNA sequence near to the Ins/Del polymorphism. Several predicted interaction regions with miRNA families were found in the *UCP2* 3’UTR sequence; however, only the hsa-miR-3668 targets the sequence where the ins/del polymorphism is located, with the 45bp Ins allele disrupting the ligation site for this miRNA, probably changing *UCP2* expression.

The Ala55Val polymorphism causes a conservative amino acid change and, until this date, there has been no indication that it causes a functional change in the protein. Therefore, assuming that the Ala55Val polymorphism is in tight LD with the -866G/A polymorphism and in moderate LD with the Ins/Del polymorphism, it is probable that this polymorphism is only reflecting the -866G/A or Ins/Del polymorphism effects on *UCP2* gene expression.

Further studies are necessary to better define if the -866G/A and Ins/Del polymorphisms have a synergistically effect on *UCP2* gene expression or if one of them has a major effect on it. Alternatively, there is a possibility that the three analyzed *UCP2* polymorphisms are not themselves responsible for the observed association with DKD, only being in LD with a still unknown functional polymorphism. Nevertheless, previous studies indicate that the -866G/A and Ins/Del polymorphisms could be directly leading to changes in *UCP2* gene expression [45,46]. Moreover, the -866A allele was reported as being associated with lower plasma total antioxidant status (increase oxidative stress) in DM patients with coronary heart disease [47], which could explain the association of the mutated haplotype containing this allele with risk for diabetic complications, including DKD.

Considering the data presented here, we therefore hypothesized that the decreased *UCP2* gene expression in kidney from carriers of the -866A/55Val/Ins haplotype might be associated with increased ROS in this tissue. Thereby, T2DM patients carrying the mutated *UCP2* haplotype could have an increased risk for DKD development since UCP2 concentration in their kidneys might not be enough to compensate the oxidative stress produced by chronic hyperglycemia. In agreement with our hypothesis, a recent study showed that genipin, an UCP2 inhibitor, dramatically boosted oxidative stress in rat renal proximal tubular cells incubated with high glucose concentrations, and this exacerbated cellular apoptosis due to an increase in caspase-3 activation [48]. In addition, He *et al*. [49] demonstrated that HUVECs (human umbilical vein endothelial cells) treated with high glucose showed an upregulation of caspase-3 and cytochrome c and the downregulation of Bcl-2 when compared to cells incubated with normal glucose concentrations. *UCP2* overexpression was able to inhibit the apoptosis of HUVECs induced by hyperglycemia. Based on these results, the authors suggested the application of UCP2 as a new protective factor for chronic diabetic complications. In contrast, Qiu *et al*. [50], reported that oral administration of genipin to diabetic mice postponed the progression of DKD, attenuating glomerular basement membrane thickness, and restoring the expression of podocin and WT1 in podocytes. They concluded that the improvement in podocyte injury was probably through the suppression of UCP2 in diabetic kidneys, which attenuated glucose-induced albumin leakage through podocytes monolayer. Thus, the role of UCP2 in kidneys still needs to be clarified.

Some factors could have interfered with the results of our case-control study. First, we cannot rule out the possibility of population stratification bias when analyzing our samples, although the number of black patients was similar in case and control groups, and frequencies of the analyzed *UCP2* polymorphisms were also similar between white and black patients. Second, we cannot exclude the possibility of a type II error when investigating the association between the analyzed polymorphisms and DKD. We had more than an 80% power (α = 0.05) to detect an OR ≥ 1.7 for the association with the -866G/A and Ala55Val polymorphisms, and we had an 80% power to detect an OR ≥ 2.0 for the Ins/Del polymorphism. Thus, we cannot rule out the possibility that these polymorphisms would be individually associated with DKD with lower ORs. The results of our *UCP2* gene expression study in kidney biopsies also should be interpreted with caution as most of our sample was constituted by patients who underwent nephrectomy, and for whom we did not have information about DKD diagnosis.

In conclusion, data reported here suggest that the *UCP2* -866A/55Val/Ins haplotype is associated with an increased risk of DKD and with a lower eGFR in T2DM patients. Furthermore, this mutated haplotype was associated with decreased *UCP2* gene expression in human kidneys. Further additional studies will be necessary to confirm the association between the *UCP2* -866A/55Val/Ins haplotype and DKD as well as to elucidate how this haplotype increases the risk of this diabetic complication. Moreover, therapeutic strategies to counteract ROS through reinforcing the action of UCP2 should be explored.
